# Characterizing phenotypic data of *Peromyscus leucopus* compared to C57BL/6J *Mus musculus* and diversity outbred (DO) *Mus musculus*

**DOI:** 10.1007/s11357-024-01175-3

**Published:** 2024-06-14

**Authors:** Lauren A. Wimer, Asia Davis-Castillo, Sofiya Galkina, Serban Ciotlos, Cavan Patterson, Leandro Prado, Maria Castro Munoz, Nicolas Martin, Sharon Epstein, Nicholas Schaum, Simon Melov

**Affiliations:** 1 Buck Institute for Research On Aging, 8001 Redwood Blvd, Novato, CA 94949 USA; 2 Astera Institute, Berkeley, CA 94710 USA

**Keywords:** Aging, Mice, Phenotyping, Diversity, Heterogeneity

## Abstract

Translational research is commonly performed in the C57B6/J mouse strain, chosen for its genetic homogeneity and phenotypic uniformity. Here, we evaluate the suitability of the white-footed deer mouse (*Peromyscus leucopus*) as a model organism for aging research, offering a comparative analysis against C57B6/J and diversity outbred (DO) *Mus musculus* strains. Our study includes comparisons of body composition, skeletal muscle function, and cardiovascular parameters, shedding light on potential applications and limitations of *P. leucopus* in aging studies. Notably, *P. leucopus* exhibits distinct body composition characteristics, emphasizing reduced muscle force exertion and a unique metabolism, particularly in fat mass. Cardiovascular assessments showed changes in arterial stiffness, challenging conventional assumptions and highlighting the need for a nuanced interpretation of aging-related phenotypes. Our study also highlights inherent challenges associated with maintaining and phenotyping *P. leucopus* cohorts. Behavioral considerations, including anxiety-induced responses during handling and phenotyping assessment, pose obstacles in acquiring meaningful data. Moreover, the unique anatomy of *P. leucopus* necessitates careful adaptation of protocols designed for *Mus musculus*. While showcasing potential benefits, further extensive analyses across broader age ranges and larger cohorts are necessary to establish the reliability of *P. leucopus* as a robust and translatable model for aging studies.

**Supplementary Information:**

The online version contains supplementary material available at 10.1007/s11357-024-01175-3.

## Introduction

The C57BL/6 J mouse is the most widely used inbred strain of laboratory mice [1–3]. The broad use of this strain has been in part due to an assumption that they have reduced phenotypic variability, largely attributed to the genetic homogeneity of the C57BL/6 J mouse. This widely held assumption has led to a general preference for inbred strains across translational research facilities. However, inbred strains such as the C57BL/6 J model may not fully represent the genetic and phenotypic diversity found in human populations, limiting their utility in translation to human health [4]. This gap has led to substantial research by researchers, such as the Interventions Testing Program (ITP), who seek to elucidate translational biology in genetically heterogeneous mouse models, such as the UM-HET3 model [5].

The diversity outbred (DO) mouse is the most genetically diverse mouse strain currently available. The DO mouse model was developed by the Complex Traits Consortium via combining five common laboratory mouse strains: A/J, C57BL/6 J, 129S1/SvlmJ, NOD/LtJ, and NZO/HlLtJ, descendants of *Mus musculus domesticus* subspecies; and three wild-derived strains: CAST/EiJ, PWK/PhJ, and WSB/EiJ, representative of the *Mus musculus* subspecies *domesticus, musculus*, and *castaneous*, respectively, in an eight-way breeding funnel [6]. The breeding of these founder strains recapitulates approximately 90% of the genetic variation seen in *Mus musculus* [7] and establishes the basis for two colonies of genetically diverse mice: the collaborative crosses (CC) and the diversity outbred (DO). Colonies of CC mice are derived through inbreeding; there are roughly 50 finished strains of CC mice which capture a representative cross-section of founder haplotypes [6]. DO mice are produced through sustained outbreeding, resulting in continued genetic heterozygosity. CC and DO mice have greater genetic diversity than those seen in human populations [8]. Through the extensive work by the Collaborative Trait Consortium, CC and DO populations demonstrated an estimated 45 million segregating polymorphisms, obtaining an advanced genetic heterogeneity that surpasses that of the human population [8]. In addition to their increased genetic variation, DO populations displayed high phenotypic variability, resulting in increased sample size, to combat weaker trait stability and unreproducible data [6]. This assertion became somewhat controversial, as one study aggregated 241 data sets to identify variance between eight inbred strains versus DO mice [9]. This study pooled assays, both behavioral and non-behavioral, ranging from changes in inflammation to femur length and nesting behavior. The results indicated that outbred DO mice are no more variable than inbred strains tested against the same parameters.

In addition to outbred mice, researchers have previously utilized nontraditional genetic model systems, such as the white-footed deer mouse (*Peromyscus leucopus*) for addressing the genetic basis of longevity and physiology [10]. *P. leucopus* are members of the Cricetidae family and diverged from the *Mus musculus* superfamily of mouselike rodents approximately 20–25 million years ago [11]. Apart from the *Mus* and *Rattus* genera, *Peromyscus* is more robustly studied than any other group of small mammals [12–14]. Several species of the *Peromyscus* genus have been used as model organisms for studies spanning from ecology and geoepidemiology to behavioral biology [15]. Contrasting *P. leucopus* and *M. musculus* was originally proposed by Sacher and Hart [16] due to the two species’ similar physical resemblance and anatomies but contrasting lifespan and health span. While *M. musculus* has a maximal lifespan potential (MLSP) of 4 years, *P. leucopus* displayed a MSLP of 7.9 years in captivity and a recorded lifespan of 8.2 years [16] and had no significant changes in luteinizing hormone (LH), estradiol, progesterone, and prolactin between 12 and 48 months, suggesting a well-maintained hypothalamic-pituitary-ovarian axis even into late age [17]. Researchers sought to better understand the mechanism for sustained longevity in *Peromyscus*, and Ungvari et al. discovered that both *P. leucopus* and *P. maniculatus* demonstrated increased DNA repair efficiency and reduced mitochondrial ROS production [18].

Despite the reported increase in longevity compared to standard laboratory mice, few researchers utilize *P. leucopus* as a viable model for aging studies. *P. leucopus* is readily available from the *Peromyscus* Genetic Stock Center. Ongoing efforts are being made towards genetic sequencing of several species of *Peromyscus*. Data on phenotypic characteristics and genetic mechanisms of longevity of laboratory-reared *P. leucopus* are widely unavailable and underrepresented. Here, we use comparative phenotyping approaches to contrast young (6 months old) *P. leucopus* and two stocks of young (6 months) *M. musculus* (inbred C57BL6/J and outbred DO) to assess *P. leucopus* as an aging model organism. Our study characterizes the phenotyped of young *P. leucopus* mice while assessing the benefits and limitations of this model as it pertains to contemporary geroscience rodent aging studies.

## Results

### Body composition testing

**Fig. 1:**
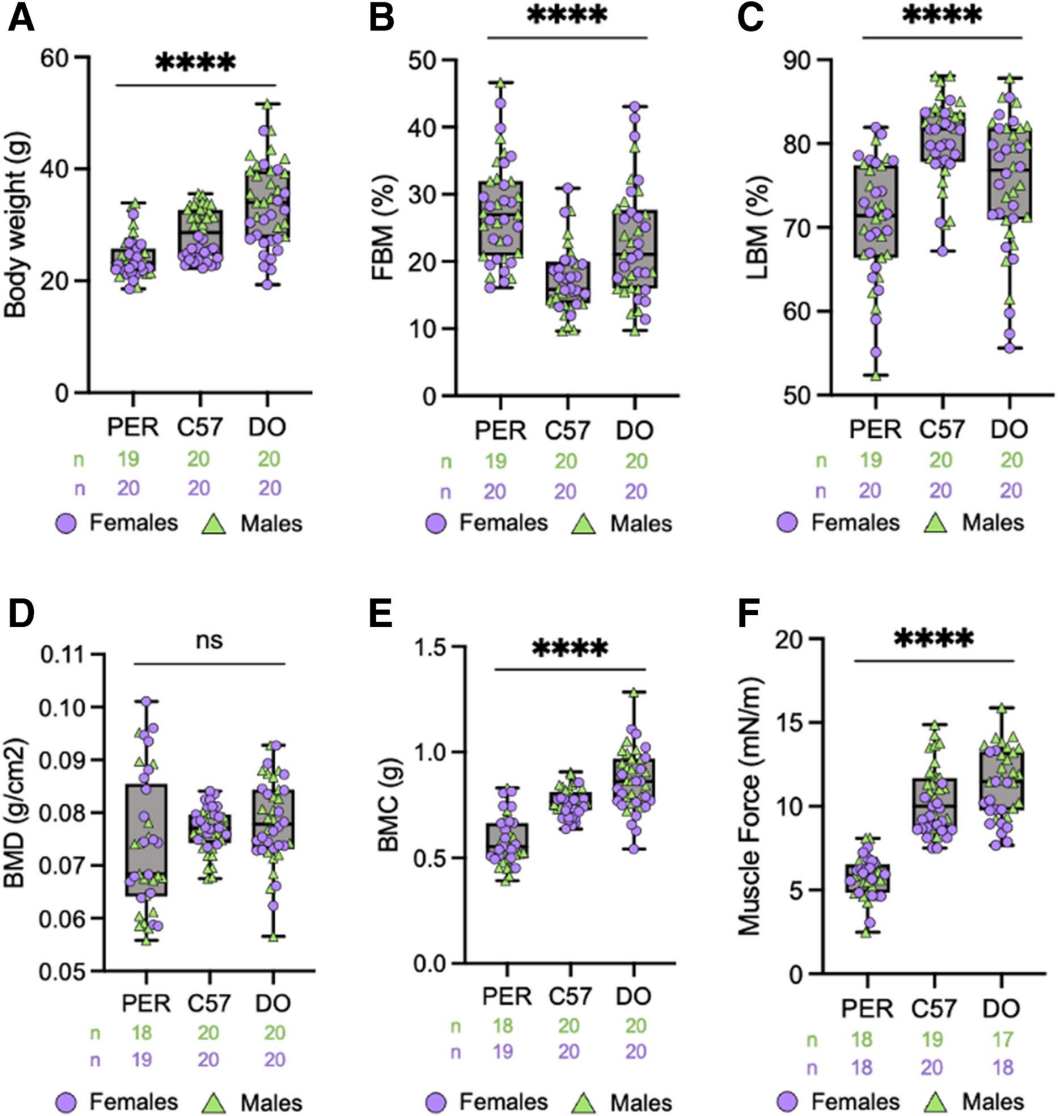
*Peromyscus leucopus* demonstrates significantly altered body composition and skeletal muscle function compared to inbred and outbred *Mus musculus* strains. **A** Total body weights of male and female *Peromyscus leucopus* (PER), C57BL6/J (C57), and diversity outbred (DO) mice, calculated by dual x-ray absorptiometry (DXA), *p* < 0.0001. **B** DXA-determined fat body mass (FBM) percentages of all three strains, *p* < 0.0001. **C** DXA-determined lean body mass (LBM) percentages of all three strains, *p* < 0.0001. **D** DXA-determined bone mineral density (BMD) of all three strains, *p* = 0.1046 (ns). **E** DXA-determined bone mineral content (BMC) of all three strains, *p* < 0.0001. **F** Recorded force (mN/m) of the *Tibealis anterior* of assayed animals following a stimulus of 125 Hz during Aurora Muscle Function Testing, *p* < 0.0001. FBM, fat body mass; LBM, lean body mass; BMD, bone mineral density; BMC, bone mineral content. Statistical analysis performed by one-way ANOVA analysis. All graphs are separated by sex in supplemental data. Significance—ns *p* > 0.05, *p < 0.05, **p < 0.005, ***p < 0.0005, and ****p < 0.0001

Dual x-ray absorptiometry (DXA) was used to directly measure fat mass and bone mass, as well as use total body weight to deduce lean body mass. One-way ANOVA analysis determined a significant difference (*p* < 0.0001) across all species/stocks. Specifically, compared to their C57 and DO counterparts, *P. leucopus* demonstrated the lowest average body weight (24.16 g, m/f) (Fig. 1A). DO mice weighed significantly more (33.86 g, m/f) than C57 mice (28.52 g, m/f), as previously reported by Chia et al. (2005). In both the C57 and DO cohorts, males weighed significantly more than their female counterparts, though this dimorphism is absent in *P. leucopus* (Supplementary Figure S1-S2). Conversely to total body weight, DXA-determined fat body mass percentage was highest in *P. leucopus* (27.51%, m/f), followed by DO (22.79%, m/f) and then C57 (17.31%, m/f, Fig. 1B). Lean body mass percentages were inversely related, with *P. leucopus* yielding the smallest values (70.57%, m/f), followed by DO (75.18%, m/f) and then C57 (80.43%, m/f, Fig. 1C). One-way ANOVA analysis of DXA-determined lean body masses determined significant (*p* < 0.0001) differences across all species and stocks. DXA-determined bone mineral density values of *P. leucopus* were the lowest of the assessed species (0.07350 g/cm^2^ m/f, Fig. 1D). Analogous to their decreased body mass and reduced bone mineral density, *P. leucopus* had the smallest bone mineral content of the assessed species (0.5784 g m/f, Fig. 1E).

### Skeletal muscle function

Muscle force was quantified by Aurora Scientific apparatus and allowed us to compare the maximal tetanic force of the hindlimb muscle, *Tibealis anterior*. The force exerted by *P. leucopus* muscle was significantly less than both C57 and DO strains when challenged by a 125 Hz stimulus, averaging 5.746 mN/m (m/f) (Fig. 1F). One-way ANOVA analysis determined significant (*p* < 0.0001) differences across all species and stocks. Further analysis of muscle function with assays such as rotarod and treadmill testing was restricted due to assay suitability for *P. leucopus*.

### Cardiac function

**Fig. 2:**
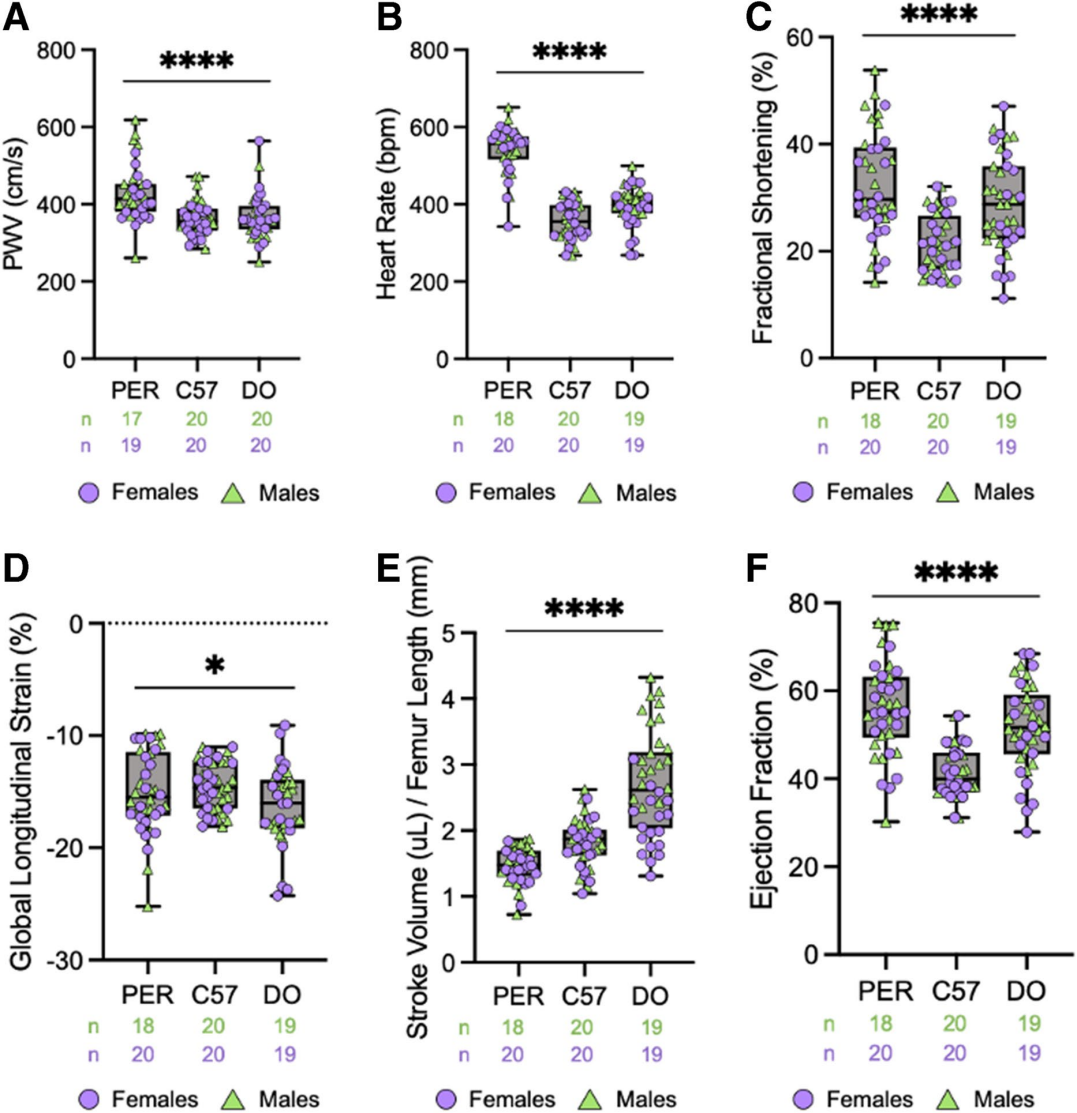
*Peromyscus leucopus* demonstrate significantly altered cardiovascular parameters compared to inbred and outbred *Mus musculus* strains. **A** Pulse wave velocity (PWV) values of male and female *Peromyscus leucopus* (PER), C57BL6/J (C57), and diversity outbred (DO) mice, calculated from the distance between the thoracic and abdominal aorta, *p* < 0.0001. **B** Heart rate (beats per minute, bpm) collected during echocardiography under 2.5% isoflurane anesthesia, *p* < 0.0001. **C** Fractional shortening calculated during echocardiography along the peristernal long axis (B-mode), *p* < 0.0001. **D** Global longitudinal strain (GLS) calculated during echocardiography along the peristernal long axis (B-mode), *p* = 0.0160. **E** Stroke volume calculated during echocardiography along the peristernal long axis (B-mode), normalized to femur lengths collected during dual x-ray absorptiometry, *p* < 0.0001. **F** Ejection fraction calculated during echocardiography along the peristernal long axis (B-mode), *p* < 0.0001. All graphs are separated by sex in supplemental data. Statistical analysis performed by one-way ANOVA analysis. Significance—ns *p* > 0.05, *p < 0.05, **p < 0.005, ***p < 0.0005, ****p < 0.0001

Intrinsic cardiac aging in the murine model closely recapitulates age-related cardiac changes in humans, including left ventricular hypertrophy, fibrosis, and diastolic dysfunction [19, 20]. Therefore, we analyzed the cardiac parameters of the *P. leucopus* model. First, we measured pulse wave velocity (PWV), the most well-established in vivo assay for measuring arterial stiffening [21–23]. This assay takes a direct measurement of the velocity at which blood pressure propagates through the circulatory system, specifically in our study, from the thoracic aorta to the abdominal aorta. Interestingly, *P. leucopus* mice demonstrated the largest pulse wave velocity (PWV) value (428.3 cm/s m/f) of the tested strains and species, suggesting stiffer arteries than C57 or DO mice (Fig. 2A). One-way ANOVA analysis determined significant (*p* < 0.0001) differences across all species and stocks. Synonymous with their increased PWV values, *P. leucopus* had the highest recorded heart rate while under 2.5% isoflurane anesthesia (540.2 bpm m/f) compared to C57 (357.6 bpm m/f) and DO (396.3 bpm m/f, Fig. 2B). Fractional shortening (FS) (the percent change in left ventricular diameter during systole) was largest in *P. leucopus* (32.28%, m/f), suggesting increased contraction capacity, though this finding is likely tied to their distinct anatomy (Fig. 2C). Global longitudinal strain (GLS), a measure of left ventricular strain was comparable among all three species (− 15.10% in *P. leucopus* (m/f), − 14.25% in C57 (m/f), and − 16.37% in DO (m/f), Fig. 2D), though still significantly different across species and stocks (one-way ANOVA, *p* = 0.0160). Despite their increased heart rate and arterial stiffness, *P. leucopus* appeared to have no more strain in the sub-endocardial fibers, suggesting an absence of cardiac strain most prone to ischemic stress and damage.

Next, we measured stroke volume (the volume of blood pumped out of the left ventricle during cardiac contraction) and ejection fraction (the percent of blood ejected from the heart with each heartbeat). As stroke volume is dependent on animal body size, all values were normalized back to individual femur lengths. Following normalization, *P. leucopus* had the smallest value for stroke volume (1.479 uL/mm m/f, Fig. 2E). While *P. leucopus* is significantly smaller than both C57 and DO strains, they had consistently longer femurs than the *Mus musculus* strains (Supplemental), possibly due to this species relying on hopping and jumping for locomotion, largely unseen in laboratory mouse stocks. Ejection fraction, non-normalized to femur length, was largest in *P. leucopus* (55.88%, m/f, Fig. 2F). Combined graphs for males and females report similar findings, but all results have been separated by sex for clarification (Supplementary Figure S3-S4). Taking the data all together, results suggest that *P. leucopus* has a more efficient heart compared to C57 and DO mouse strains. Another possibility is that normalization factors generally used with *Mus musculus* models may not be appropriate for the *P. leucopus* model, and alternative methods should be taken into consideration during both data acquisition and interpretation.

## Discussion

Here, we report phenotypic differences found between the wild white-footed deer mouse (*P. leucopus*) and inbred (C57) and outbred (DO*) Mus musculus* and assess its ease of use for aging studies. While *P. leucopus* appear physically similar to *Mus musculus*, they demonstrate significant anatomical differences in their bone size and length, lean muscle mass, eye size, and internal organ size [24, 25]. Our data reflected that the body composition of *P. leucopus* was more heavily made up of fat mass, suggesting a unique metabolism within this species compared to *Mus musculus*. It is known that energy expenditure (EE) is largely driven by lean body mass. However, fat mass also makes a large contribution towards EE [26]. It is possible that fat metabolism plays a role in the large differential in maximal lifespans, and further metabolic analyses are required to explore this possibility in this model.

Interestingly, the cardiovascular results of *P. leucopus* seem indicative of a damaged cardiovascular system, when compared to typical results seen in *Mus musculus*. However, simplistic cross-sectional comparisons among different species, without extensive normalization appropriate for each species, may adversely affect such measurements, complicating such an interpretation.

First, the *P. leucopus* cohort demonstrated an increased heart rate (bpm) and elevated pulse wave velocity (PWV). The PWV assay supports a well-known concept that arterial stiffness is an intrinsic characteristic of aging [27]. Specifically, there is considerable data to support that aortic stiffness increases with age and precedes the development of cardiovascular diseases, such as hypertrophy and hypertension [27]. The mechanism through which aortic stiffening occurs is complex and includes changes in the extracellular matrix (ECM) as well as vascular smooth muscle cells, as opposed to endothelial cells. This assay is widely used in human studies, and PWV increases with increasing heart rate in humans. Arterial stiffness is a complex physiological trait, and detailed assessment requires measurement of additional parameters, unexplored in our study. Future studies would be beneficial to determine whether *P. leucopus* demonstrates pathological symptoms of aortic stiffening, such as the presence of collagen I, III, and IV fibers within cardiac tissue, as well as biomarkers of cardiac inflammation such as CRP, klotho, and aldosterone with increasing age [28].

The increased in vivo PWV values of the *P. leucopus* model were contradictive to their global longitudinal strain (GLS) results. Recently, left ventricular GLS, as assessed by m-mode echocardiography, has been accepted as a measure of early cardiac dysfunction. A 2017 study by Bell et al. analyzed 2495 human participants free of cardiovascular disease and found increased aortic stiffness to be associated with worsening global longitudinal strain [29]. This data supports a hypothesis that *P. leucopus* mice retain different “normal” ranges for cardiovascular assays, such as the PWV, and highlights the need for further exploration of *P. leucopus* cardio-vasculature.

Echocardiograph-acquired stroke volumes were normalized to the femur length of each animal in our study to interpret cardiac output. This normalization was chosen due to its use in both *Mus musculus* as well as *Homo sapiens* (human) M-mode echocardiography data analysis. Previously published articles interpreted a *z*-score of cardiac output with the independent variable being either femur length or biparietal diameter [30]. In our study, the normalized stroke volume of *P. leucopus* mice demonstrated a highly efficient heart, with significantly increased cardiac output. Our analysis does not consider anatomical changes between the *P. leucopus* mouse and the *Mus musculus* stocks and does not account for differential bone sizes due to unique locomotion in *P. leucopus*.

Vascular aging is a widely studied field of aging and represents a collection of histological and functional changes in blood vessels with age. Clinical assessment of vascular aging has given rise to specific paradigms and hallmarks, such as increased arterial wall thickness, dysfunctional vasodilation, and decreased endothelial progenitor cells [31]. Phenotypic assays such as echocardiography and pulse wave velocity (PWV) have paved the way for researchers to measure some of these hallmarks in vivo, advancing further discovery for early interventions for cardiomyopathies, and there are dozens of applicable ranges for varying strains and stocks of *Mus musculus* and *Rat rattus*. With the underrepresentation of phenotypic data from *P. leucopus*, it is difficult to draw meaningful conclusions from the cardiovascular measures in our study at a single timepoint. Further studies are needed to gain insight into the possibly novel cardiovascular system of the *P. leucopus* model, opening further opportunities in combatting age-associated cardiovascular decline previously unexplored in other murine models.

## Limitations

In our studies with *P. leucopus*, we uncovered both behavioral and physiological concerns for multiple phenotyping assays, which should be considered prior to using these mice as an aging model. First, much of the *P. leucopus* cohort was highly agitated and prone to anxious behavior. This behavior was exacerbated when animals were handled or removed from their home cage for any extended period. For example, metabolic cage data acquired from single-housed *P. leucopus* demonstrated these animals’ refused food and water, and minimally moved, and lost upwards of 25% of their total body weight during beta testing for measuring metabolic rate in metabolic cages (data not shown). This limited our ability to acquire meaningful data from metabolic caging of these animals. The reason behind the single-housed anxiety of our *Peromyscus leucopus* cohort is not well understood. One potential reason may be that these mice were not bred for captivity. However, the mice used in our study are derived from 38 animals, originally caught between 1982 and 1985, and bred and housed over the last 40 years in sustained captivity (Peromyscus Genetic Stock Center, North Carolina). We note that acclimation of single-housed mice was carried out prior to metabolic cage testing. It has been previously reported in a cohort of BALB/c mice that metabolic cages induce stress, regardless of acclimation time, due to the non-enriched environment [32]. It is possible that *P. leucopus* have increased sensitivity to environmental stressors, rendering them unsuitable for singly housed assays like metabolic cages.

A second limitation found within our studies of the *P. leucopus* mouse model is their high level of ambulatory activity, resulting in limitations for behavioral assays. Aging studies often encompass deficits in memory, cognition, and motor coordination, requiring specialized equipment and assay protocols. We determined that *P. leucopus* is highly active and easily escapes most caging apparatuses and assay equipment. Historically, behavioral assays such as the Y-maze and the elevated plus maze have been designed for testing laboratory, often including platforms that laboratory mice would not jump from, or low walls that mice would not leap over. Unfortunately, these designs are incompatible with *P. leucopus*.

Further limitations of our study pertain to the anatomical differences of *P. leucopus* compared to *Mus musculus*. All the phenotyping assays we have outlined were optimized for the analysis of *Mus musculus* and widely untested in other murine models. Specifically, the Aurora muscle force apparatus protocol was established to assess muscle fatigue in *Mus musculus*. It should be noted that the muscle function protocol and stimuli were not adjusted for *P. leucopus* and that their individual muscle anatomy may play a role in their reduced recorded muscle force. Additionally, though the PWV apparatus (Scintica) has been used with neonatal and newborn *Mus musculus* studies [33] and is assumed to work for animals with reduced arterial size and reduced body size, there is no data available for *P. leucopus*. Finally, molecular analysis of tissues from *P. leucopus* remains elusive as they are non-homologous with *Mus musculus* and require specialized equipment and reagents for antibody or sequence-based testing.

In summary*,* our study supports *P. leucopus* as having the potential to be a meaningful mammalian model for aging studies in areas such as body composition and cardiovascular health, particularly given the reported longevity of the species, almost triple that of conventional lab mice.

## Methods and materials

### Mice

Studies included the use of male and virgin females of two *Mus musculus* strains and stocks, and one species of *Peromyscus leucopus* (white-footed deer mouse), all aged 6 months. *Mus musculus* strains and stocks include the C57BL6/J (#006664, Jackson Laboratories) strain and diversity outbred (DO) (#009736, Jackson Laboratories) stock. *P. leucopus* mice come from the Peromyscus Stock Center (University of South Carolina). All *Mus musculus* mice were communally housed and aged-matched with ad libitum access to water and diet (Envigo Teklad #2918) in a pathogen and temperature-controlled room with a 12 h light–dark cycle beginning at 06:00 AM. All procedures were conducted in accordance with NIH Guidelines for Care and Use of Animals and were approved by the Institutional Animal Care and Use Committees at Buck Institute for Research on Aging.

All *P. leucopus* were communally housed and age-matched with ad libitum access to water and diet (Envigo Teklad #2918) in an isolated pathogen and temperature-controlled room with a 12-h light–dark cycle beginning at 06:00 AM. Upon arrival at the Buck Institute, *P. leucopus* mice were housed in cages of four mice per cage and quarantined at the Buck facility for 3 months. Following their 3-month quarantine, all mice passed parasitology and serology testing and regular handling began with vivarium and veterinarian staff to ensure proper care and handling. All colony management and experimental procedures were conducted in accordance with USDA regulation, NIH Guidelines for Care and Use of Animals, and were approved by the Institutional Animal Care and Use Committees at Buck Institute for Research on Aging.

### Body composition testing

Dual-energy x-ray absorptiometry (DEXA) scans were used to quantify body composition in anesthetized (2% isoflurane) immobilized mice by InAlyzer2S (Micro Photonics).

### Muscle function testing

Muscle function was recorded by the Aurora Scientific In Vivo Isolated Muscle System for Mice. Mice were briefly anesthetized with 2.5% isoflurane and subjected to stimulation of their hind limb muscle. Once anesthetized, mice are secured to a heating pad, and their rear leg is secured into a foot plate attached to an electric motor. Two needle electrodes are inserted through the skin behind the knee to deliver voltage and measure muscle contraction.

### Pulse wave velocity testing

Pulse wave velocity recordings were collected by Indus Doppler Systems, while mice were anesthetized (2.5% isoflurane) immobilized. Two fixed probes were placed at the thoracic aorta and the abdominal aorta to measure pulse waves, and the distance between the probes was measured by caliper to determine velocity.

### Echocardiography testing

Echocardiography parameters were collected using VisualSonics Vevo 770 in anesthetized (2.5% isoflurane) immobilized mice. Ultrasound images of the lateral ventricle were collected by both long-axis and short-axis visualization. Analysis was performed by Vevo Lab workstation application.

### Quantification and statistical analysis

Statistical details of experiments can be found in the figure legends. All data are expressed as mean ± SEM as indicated in the figure legends. Statistical tests were selected based on appropriate assumptions with respect to data distribution and variance characteristics. For normally distributed data, statistical significance was determined using one-way ANOVA. All statistical analyses were performed using GraphPad Prism. Significant differences are indicated as follows: ^∗^*p* ≤ 0.05, ^∗∗^*p* ≤ 0.005, ^∗∗∗^*p* ≤ 0.0005, and ^∗∗∗∗^*p* < 0.0001.

### Supplementary Information

Below is the link to the electronic supplementary material.

Supplementary file1 (DOCX 314 KB)

## References

[1] Beck JA, et al. Genealogies of mouse inbred strains. Nat Genet. 2000;24(1):23–5. 10615122 10.1038/71641

[2] Myers DD, Meier H. Prevalence of murine C-type RNA virus group specific antigen in inbred strains of mice. Life Sci. 1970;9(19):1071–80. 10.1016/0024-3205(70)90016-0 4320181

[3] Festing MF, Blackmore DK. Life span of specified-pathogen-free (MRC category 4) mice and rats. Lab Anim. 1971;5(2):179–92. 5166568 10.1258/002367771781006564

[4] Tyshkovskiy A, et al. Identification and application of gene expression signatures associated with lifespan extension. Cell Metab. 2019;30(3):573-593.e8. 31353263 10.1016/j.cmet.2019.06.018 PMC6907080

[5] Harrison DE, et al. 17-a-estradiol late in life extends lifespan in aging UM-HET3 male mice; nicotinamide riboside and three other drugs do not affect lifespan in either sex. Aging Cell. 2021;20(5):e13328. 33788371 10.1111/acel.13328 PMC8135004

[6] Saul MC, et al. High-diversity mouse populations for complex traits. Trends Genet. 2019;35(7):501–14. 31133439 10.1016/j.tig.2019.04.003 PMC6571031

[7] Roberts A, et al. The polymorphism architecture of mouse genetic resources elucidated using genome-wide resequencing data: implications for QTL discovery and systems genetics. Mamm Genome. 2007;18(6–7):473–81. 17674098 10.1007/s00335-007-9045-1 PMC1998888

[8] Keane TM, et al. Mouse genomic variation and its effect on phenotypes and gene regulation. Nature. 2011;477(7364):289–94. 21921910 10.1038/nature10413 PMC3276836

[9] Tuttle AH, et al. Comparing phenotypic variation between inbred and outbred mice. Nat Methods. 2018;15(12):994–6. 30504873 10.1038/s41592-018-0224-7 PMC6518396

[10] Long PN, et al. The utility of a closed breeding colony of Peromyscus leucopus for dissecting complex traits*.* Genetics. 2022;221(1). 10.1093/genetics/iyac026 PMC9071557 35143664

[11] Steppan S, Adkins R, Anderson J. Phylogeny and divergence-date estimates of rapid radiations in muroid rodents based on multiple nuclear genes. Syst Biol. 2004;53(4):533–53. 15371245 10.1080/10635150490468701

[12] King JA. Intra and interspecific conflict of Mus and Peromyscus. Ecology. 1957;38:355–7. 10.2307/1931697

[13] King JA. Biology of Peromyscus (Rodentia). Am Soc Mammologists. 1968.

[14] Kirkland GL, Layne JN. Advances in the study of Peromyscus (Rodentia)*.* J Mammal. 1989;71(3). 10.2307/1381970

[15] Earnest R, et al. Survey of white-footed mice (*Peromyscus leucopus*) in Connecticut, USA reveals low SARS-CoV-2 seroprevalence and infection with divergent betacoronaviruses. npj Viruses 2023;**1**(1). 10.1038/s44298-023-00010-4 10.1038/s44298-023-00010-4 PMC11721133 40295640

[16] Sacher GA. Longevity, aging and comparative cellular and molecular biology of the house mouse, *Mus musculus*, and the white-footed mouse, *Peromyscus leucopus.* Birth Defects Orig Artic Ser. 1978;14(1):71–96. 343832

[17] Steger RW, et al. Effects of advancing age on the hypothalamic-pituitary-ovarian axis of the female white-footed mouse (Peromyscus leucopus). Exp Aging Res. 1980;6(4):329–39. 7000517 10.1080/03610738008258368

[18] Ungvari Z, et al. Testing hypotheses of aging in long-lived mice of the genus Peromyscus: association between longevity and mitochondrial stress resistance, ROS detoxification pathways, and DNA repair efficiency. Age (Dordr). 2008;30(2–3):121–33. 19424862 10.1007/s11357-008-9059-y PMC2527628

[19] de Lucia C, et al. Echocardiographic strain analysis for the early detection of left ventricular systolic/diastolic dysfunction and dyssynchrony in a mouse model of physiological aging. J Gerontol A Biol Sci Med Sci. 2019;74(4):455–61. 29917053 10.1093/gerona/gly139 PMC6417453

[20] Dai DF, Rabinovitch PS. Cardiac aging in mice and humans: the role of mitochondrial oxidative stress. Trends Cardiovasc Med. 2009;19(7):213–20. 20382344 10.1016/j.tcm.2009.12.004 PMC2858758

[21] Gioscia-Ryan RA, et al. Lifelong voluntary aerobic exercise prevents age- and Western diet- induced vascular dysfunction, mitochondrial oxidative stress and inflammation in mice. J Physiol. 2021;599(3):911–25. 33103241 10.1113/JP280607 PMC7856030

[22] Brunt VE, et al. Trimethylamine-N-oxide promotes age-related vascular oxidative stress and endothelial dysfunction in mice and healthy humans. Hypertension. 2020;76(1):101–12. 32520619 10.1161/HYPERTENSIONAHA.120.14759 PMC7295014

[23] Gioscia-Ryan RA, et al. Mitochondria-targeted antioxidant therapy with MitoQ ameliorates aortic stiffening in old mice. J Appl Physiol (1985). 2018;124(5):1194–202. 29074712 10.1152/japplphysiol.00670.2017 PMC6008077

[24] Shupe JM, et al. The eye of the laboratory mouse remains anatomically adapted for natural conditions. Brain Behav Evol. 2006;67(1):39–52. 16219997 10.1159/000088857 PMC2582157

[25] Sun Y, et al. Peromyscus leucopus mice: a potential animal model for haematological studies. Int J Exp Pathol. 2014;95(5):342–50. 25116892 10.1111/iep.12091 PMC4209926

[26] Kaiyala KJ, et al. Identification of body fat mass as a major determinant of metabolic rate in mice. Diabetes. 2010;59(7):1657–66. 20413511 10.2337/db09-1582 PMC2889765

[27] De Moudt S, et al. Progressive aortic stiffness in aging C57Bl/6 mice displays altered contractile behaviour and extracellular matrix changes. Commun Biol. 2022;5(1):605. 35710942 10.1038/s42003-022-03563-x PMC9203497

[28] Angoff R, Mosarla RC, Tsao CW. Aortic stiffness: epidemiology, risk factors, and relevant biomarkers. Front Cardiovasc Med. 2021;8:709396. 34820427 10.3389/fcvm.2021.709396 PMC8606645

[29] Bell V, et al. Relations between aortic stiffness and left ventricular mechanical function in the community. J Am Heart Assoc. 2017;6(1). 10.1161/JAHA.116.004903 10.1161/JAHA.116.004903 PMC5523643 28069573

[30] Moon-Grady AJ, et al. Guidelines and recommendations for performance of the fetal echocardiogram: an update from the American society of echocardiography. J Am Soc Echocardiogr. 2023;36(7):679–723. 37227365 10.1016/j.echo.2023.04.014

[31] Li A, et al. Vascular aging: assessment and intervention. Clin Interv Aging. 2023;18:1373–95. 37609042 10.2147/CIA.S423373 PMC10441648

[32] Kalliokoski O, et al. Mice do not habituate to metabolism cage housing–a three week study of male BALB/c mice. PLoS ONE. 2013;8(3):e58460. 23505511 10.1371/journal.pone.0058460 PMC3591308

[33] Fitzsimons LA, et al. Noninvasive electrocardiography in the perinatal mouse*.* J Vis Exp. 2020;(160). 10.3791/61074 10.3791/61074 32597855

