# Supplementary material for: Characterizing phenotypic data of Peromyscus leucopus compared to C57BL/6J Mus musculus and diversity outbred (DO) Mus musculus
Source: GeroScience. 2024 Jun 14;46(5):4647–56. doi: 10.1007/s11357-024-01175-3 (PMC11335981; doi:10.1007/s11357-024-01175-3)
Supplement: Supplementary file 1 — Supplementary file1 (DOCX 314 KB) [file 11357_2024_1175_MOESM1_ESM.docx]

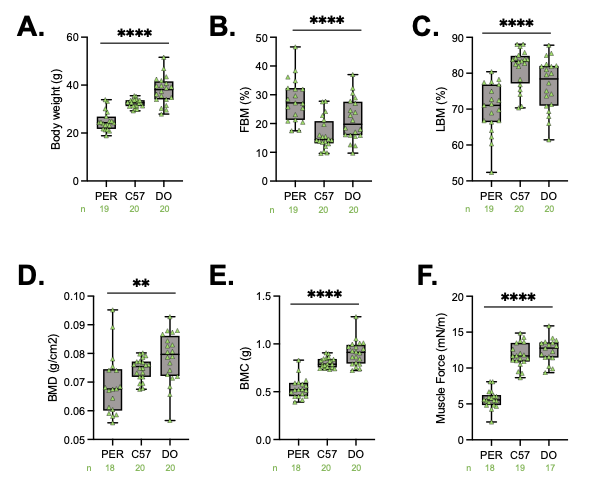


**Supplemental Figure S1: Male *Peromyscus leucopus* demonstrate significantly altered body composition and skeletal muscle function compared to inbred and outbred male *Mus musculus* strains.** A) total body weights of male *Peromyscus leucopus* (PER), C57BL6/J (C57), and diversity outbred (DO) mice, calculated by dual x-ray absorptiometry (DXA). B) DXA-determined fat body mass (FBM) percentages of all three strains. C) DXA-determined lean body mass (LBM) percentages of all three strains. D) DXA-determined bone mineral density (BMD) of all three strains E) DXA-determined bone mineral content (BMC) of all three strains. F) Recorded force (mN/m) of the *Tibealis anterior* of assayed animals following a stimulus of 125 Hz during Aurora Muscle Function Testing. FBM = fat body mass, LBM = lean body mass, BMD = bone mineral density, BMC = bone mineral content. Statistical analysis performed by one-way ANOVA analysis. Significance – ns p.> 0.05, * p < 0.05, ** p < 0.005, *** p < 0.0005, **** p < 0.0001


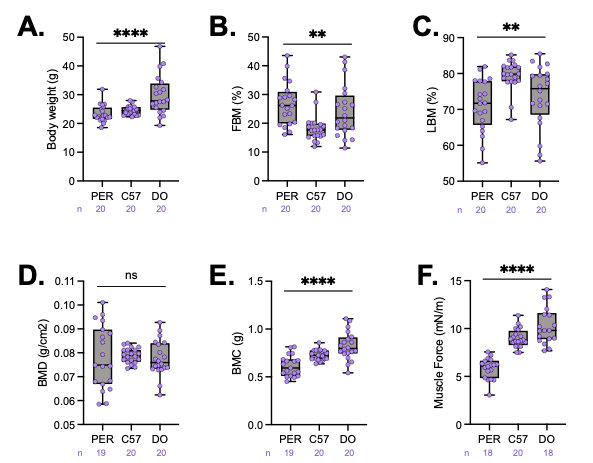


**Supplemental Figure S2: Female *Peromyscus leucopus* demonstrate significantly altered body composition and skeletal muscle function compared to inbred and outbred female *Mus musculus* strains.** A) total body weights of female *Peromyscus leucopus* (PER), C57BL6/J (C57), and diversity outbred (DO) mice, calculated by dual x-ray absorptiometry (DXA). B) DXA-determined fat body mass (FBM) percentages of all three strains. C) DXA-determined lean body mass (LBM) percentages of all three strains. D) DXA-determined bone mineral density (BMD) of all three strains E) DXA-determined bone mineral content (BMC) of all three strains. F) Recorded force (mN/m) of the *Tibealis anterior* of assayed animals following a stimulus of 125 Hz during Aurora Muscle Function Testing. FBM = fat body mass, LBM = lean body mass, BMD = bone mineral density, BMC = bone mineral content. Statistical analysis performed by one-way ANOVA analysis. Significance – ns p.> 0.05, * p < 0.05, ** p < 0.005, *** p < 0.0005, **** p < 0.0001


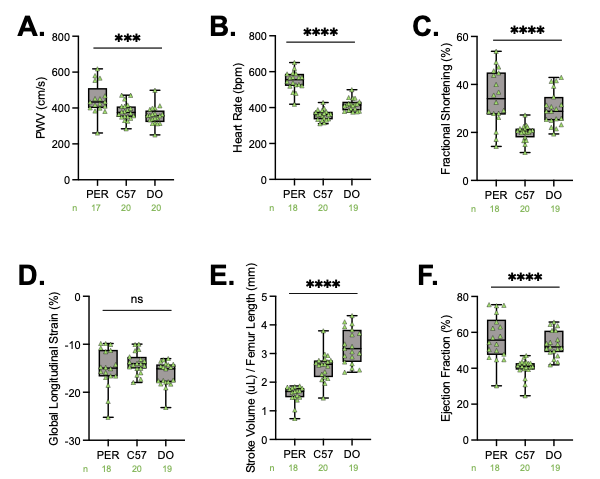


**Supplemental Figure S3: Male *Peromyscus leucopus* demonstrate significantly altered cardiovascular parameters compared to inbred and outbred male *Mus musculus* strains.** A) Pulse wave velocity (PWV) values of male *Peromyscus leucopus* (PER), C57BL6/J (C57), and diversity outbred (DO) mice, calculated from the distance between the thoracic and abdominal aorta. B) Heart rate (beats per minute, bpm) collected during echocardiography under 2.5% isoflurane anesthesia. C) Fractional shortening calculated during echocardiography along the peristernal long axis (B-mode). D) Global longitudinal strain (GLS) calculated during echocardiography along the peristernal long axis (B-mode). E) Stroke volume calculated during echocardiography along the peristernal long axis (B-mode), normalized to femur lengths collected during dual x-ray absorptiometry. F) Ejection fraction calculated during echocardiography along the peristernal long axis (B-mode). Statistical analysis performed by one-way ANOVA analysis. Significance – ns p.> 0.05, * p < 0.05, ** p < 0.005, *** p < 0.0005, **** p < 0.0001


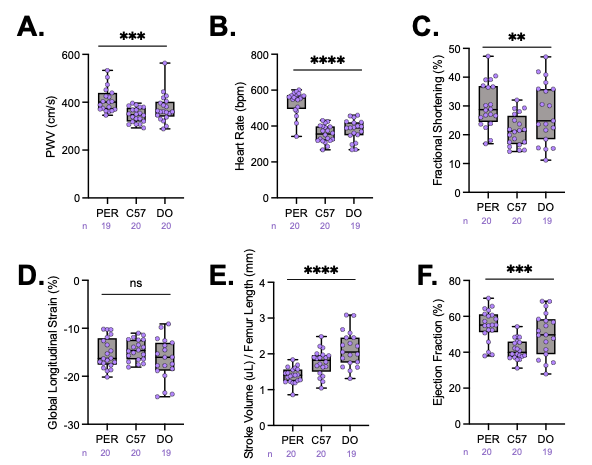


**Supplemental Figure S4: Female *Peromyscus leucopus* demonstrate significantly altered cardiovascular parameters compared to inbred and outbred female *Mus musculus* strains.** A) Pulse wave velocity (PWV) values of female *Peromyscus leucopus* (PER), C57BL6/J (C57), and diversity outbred (DO) mice, calculated from the distance between the thoracic and abdominal aorta. B) Heart rate (beats per minute, bpm) collected during echocardiography under 2.5% isoflurane anesthesia. C) Fractional shortening calculated during echocardiography along the peristernal long axis (B-mode). D) Global longitudinal strain (GLS) calculated during echocardiography along the peristernal long axis (B-mode). E) Stroke volume calculated during echocardiography along the peristernal long axis (B-mode), normalized to femur lengths collected during dual x-ray absorptiometry. F) Ejection fraction calculated during echocardiography along the peristernal long axis (B-mode). Statistical analysis performed by one-way ANOVA analysis. Significance – ns p.> 0.05, * p < 0.05, ** p < 0.005, *** p < 0.0005, **** p < 0.0001
